# Supplementary material for: C. elegans LIN-66 mediates EIF-3/eIF3-dependent protein translation via a cold-shock domain
Source: Life Sci Alliance. 2024 Jun 17;7(9):e202402673. doi: 10.26508/lsa.202402673 (PMC11184513; doi:10.26508/lsa.202402673)
Supplement: Supplementary file 1 [file LSA-2024-02673_TableS1.docx]

**Table S1: Strains and genotypes.**

| strain | genotype | transgene/allele description | observed results |
| --- | --- | --- | --- |
| N2 | *+* | *RRID:CGC_N2* | *see Methods: Mapping of lin-66(ju1661)* |
| MT6241 | *acr-2*(*n2420*) X | Described in Jospin et al (2009); also see Table S2 (G925A, Val309Met,) | Fig. 1B |
| CZ27230 | *lin-66*(*ju1661*) IV | Nucleotide transition: G2052A at splice acceptor | Fig. 1B |
| CZ21759 | *eif-3.G*(*ju807*) II ; *acr-2*(*n2420*) X | *eif-3.G*(*ju807*) is described in Blazie et al. (2021), also see Table S2. | Fig. 1B |
| CZ27762 | *lin-66*(*ju1661*) IV ; *acr-2*(*n2420*) X |  | Fig. 1B |
| CZ26710 | *eif-3.G*(*ju807*) II; *lin-66*(*ju1661*) IV; *acr-2*(*n2420*) X | Original isolate of *ju1661* from the genetic screen (see Methods) | *see Methods: Mapping of lin-66(ju1661)* |
| CZ26711 | *eif-3.G*(*ju807*) II; *lin-66*(*ju1661*) IV; *acr-2*(*n2420*) X | Outcrossed *ju1661* (see Methods) | Fig. 1B |
| CZ28971 | *lin-66*(ku423) IV/nT1(qIs51) IV; V | Balanced *lin-66*(*0*) |  |
| CZ28952 | *lin-66*(ku423) IV/nT1(qIs51) IV; V; *acr-2*(*n2420*) |  | Fig. 1B |
| CZ28953 | *eif-3.G(ju807); lin-66*(ku423) IV/nT1(qIs51) IV; V; *acr-2(n2420)* |  | Fig. 1B |
| CZ27510 | *eif-3.G*(*ju807*) II; *lin-66*(*ju1661*) IV; *acr-2*(*n2420*); *juEx8032[Plin-66::lin-66gDNA]* |  | Fig. 1B |
| CZ27511 | *eif-3.G*(*ju807*) II; *lin-66*(*ju1661*) IV; *acr-2*(*n2420*); *juEx8033[Plin-66::lin-66gDNA]* |  | Fig. 1B |
| OP433 | *unc-119*(*tm4063*) III ; *wgIs433[hlh-30::GFP(fosmid)]* | RRID: CGC_OP433 | Fig. 2 |
| CZ28145 | *eif-3.g(ju807) II; unc-119*(*tm4063*) III ; *wgIs433[hlh-30::GFP(fosmid)]* |  | Fig. 2 |
| CZ27913 | *acr-2(gf) X; unc-119*(*tm4063*) III ; *wgIs433[hlh-30::GFP(fosmid)]* |  | Fig. 2 |
| CZ27914 | *eif-3.g(ju807)* II*; unc-119*(*tm4063*) III ; *acr-2*(*gf*) X; *wgIs433[hlh-30::GFP(fosmid)]* |  | Fig. 2 |
| CZ29603 | *lin-66(ju1661) IV; unc-119*(*tm4063*) III ; *wgIs433[hlh-30::GFP(fosmid)]* |  | Fig. 2 |
| CZ28775 | *lin-66(ju1661) IV; acr-2(gf) X; unc-119*(*tm4063*) III ; *wgIs433[hlh-30::GFP(fosmid)]* |  | Fig. 2 |
| CZ28883 | *lin-66(ju1661) IV; eif-3.g(ju807)* II*; unc-119*(*tm4063*) III ; *acr-2*(*gf*) X; *wgIs433[hlh-30::GFP(fosmid)]* |  | Fig. 2 |
| CZ29272 | *eif-3.G*(*ju807*) II; *lin-66*(*ju1661*) IV; *acr-2*(*n2420*); *juEx8184[Punc-17B-lin-66C-GFP]* |  | Fig. 1C |
| CZ29273 | *eif-3.G*(*ju807*) II; *lin-66*(*ju1661*) IV; *acr-2*(*n2420*); *juEx8185[Punc-17B-lin-66C-GFP]* |  | Fig. 1C |
| CZ29276 | *eif-3.G*(*ju807*) II; *lin-66*(*ju1661*) IV; *acr-2*(*n2420*); *juEx8186*[*Punc-17B-lin-66A-GFP*] |  | Fig. 1C |
| CZ29277 | *eif-3.G*(*ju807*) II; *lin-66*(*ju1661*) IV; *acr-2*(*n2420*); *juEx8187*[*Punc-17B-lin-66A-GFP*] |  | Fig. 1C |
| CZ29280 | *eif-3.G*(*ju807*) II; *lin-66*(*ju1661*) IV; *acr-2*(*n2420*); *juEx8190[Plin-66-lin-66C-GFP*] |  | Fig. 4 |
| CZ29288 | *eif-3.G*(*ju807*) II; *lin-66*(*ju1661*) IV; *acr-2*(*n2420*); *juEx8191[Plin-66-lin-66C-GFP*] |  | Fig. 4 |
| CZ29395 | *eif-3.G*(*ju807*) II; *lin-66*(*ju1661*) IV; *acr-2*(*n2420*); *juEx8240[Plin-66-lin-66C(∆10-44)-GFP*] |  | Fig. 4 |
| CZ29396 | *eif-3.G*(*ju807*) II; *lin-66*(*ju1661*) IV; *acr-2*(*n2420*); *juEx8241[Plin-66-lin-66C(∆10-44)-GFP*] |  | Fig. 4 |
| CZ29298 | *eif-3.G*(*ju807*) II; *lin-66*(*ju1661*) IV; *acr-2*(*n2420*); *juEx8194[Plin-66-lin-66C(∆421-554)-GFP*] |  | Fig. 4 |
| CZ29299 | *eif-3.G*(*ju807*) II; *lin-66*(*ju1661*) IV; *acr-2*(*n2420*); *juEx8195[Plin-66-lin-66C(∆421-554)-GFP*] |  | Fig. 4 |
| CZ29397 | *eif-3.G*(*ju807*) II; *lin-66*(*ju1661*) IV; *acr-2*(*n2420*); *juEx8242[Plin-66-lin-66C(∆10-44 + ∆421-554)-GFP*] |  | Fig. 4 |
| CZ29398 | *eif-3.G*(*ju807*) II; *lin-66*(*ju1661*) IV; *acr-2*(*n2420*); *juEx8243[Plin-66-lin-66C(∆10-44 + ∆421-554)-GFP*] |  | Fig. 4 |
| CZ29301 | *eif-3.G*(*ju807*) II; *lin-66*(*ju1661*) IV; *acr-2*(*n2420*); *juEx8197[Plin-66-lin-66C((∆116-367)-GFP*] |  | Fig. 4 |
| CZ29302 | *eif-3.G*(*ju807*) II; *lin-66*(*ju1661*) IV; *acr-2*(*n2420*); *juEx8198[Plin-66-lin-66C((∆116-367)-GFP*] |  | Fig. 4 |
| CZ29349 | *eif-3.G*(*ju807*) II; *lin-66*(*ju1661*) IV; *acr-2*(*n2420*); *juEx8206[Plin-66-lin-66C(∆298-367)-GFP*] |  | Fig. 4 |
| CZ29350 | *eif-3.G*(*ju807*) II; *lin-66*(*ju1661*) IV; *acr-2*(*n2420*); *juEx8207[Plin-66-lin-66C(∆298-367)-GFP*] |  | Fig. 4 |
| CZ29353 | *eif-3.G*(*ju807*) II; *lin-66*(*ju1661*) IV; *acr-2*(*n2420*); *juEx8220[Plin-66-lin-66C(∆10-172)-GFP*] |  | Fig. 4 |
| CZ29354 | *eif-3.G*(*ju807*) II; *lin-66*(*ju1661*) IV; *acr-2*(*n2420*); *juEx8221[Plin-66-lin-66C(∆10-172)-GFP*] |  | Fig. 4 |
| CZ29393 | *eif-3.G*(*ju807*) II; *lin-66*(*ju1661*) IV; *acr-2*(*n2420*); *juEx8238[Plin-66-lin-66C(∆2-90-∆387-624)-GFP*] |  | Fig. 4 |
| CZ29394 | *eif-3.G*(*ju807*) II; *lin-66*(*ju1661*) IV; *acr-2*(*n2420*); *juEx8239[Plin-66-lin-66C(∆2-90-∆387-624)-GFP*] |  | Fig. 4 |
| CZ29347 | *lin-66*(*ju1934*) IV | Intermediate product of GFP knock-in to tag lin-66 at C-terminus | Fig. 3 |
| CZ29973 | *lin-66*(*ju1934*) IV; *acr-2*(*n2420*) X |  | Fig. 3 |
| CZ29975 | *eif-3.G*(*C130Y*) II; *lin-66*(*ju1934*) IV |  | Fig. 3 |
| CZ29976 | *eif-3.G*(*C130Y*) II; *lin-66*(*ju1934*) IV; *acr-2*(*n2420*) X |  | Fig. 3 |
| CZ29376 | *juSi395[mKate2::eif-3.G] I; lin-66*(*ju1934*) IV |  | Fig. 3C |
| CZ29408 | *juSi395[mKate2::eif-3.G] I; lin-66*(*ju1934*) IV; *acr-2*(*n2420*) X |  | Fig. 3C |
| CZ29607 | *eif-3.G*(*ju807*) II; *lin-66*(*ju1661*) IV; *acr-2*(*n2420*); *juEx8267[Plin-66-lin-66C(CSD*)-GFP*] | CSD*- mutated LIN-66 cold-shock domain with following amino acid substitution: G116A, L117A, I118S, S127A, F128A, Q129S | Fig. 5C and 5D |
| CZ29608 | *eif-3.G*(*ju807*) II; *lin-66*(*ju1661*) IV; *acr-2*(*n2420*); *juEx8268[Plin-66-lin-66C(CSD*)-GFP*] | CSD*- mutated LIN-66 cold-shock domain with following amino acid substitution: G116A, L117A, I118S, S127A, F128A, Q129S | Fig. 5C and 5D |
