# Supplementary material for: C. elegans LIN-66 mediates EIF-3/eIF3-dependent protein translation via a cold-shock domain
Source: Life Sci Alliance. 2024 Jun 17;7(9):e202402673. doi: 10.26508/lsa.202402673 (PMC11184513; doi:10.26508/lsa.202402673)
Supplement: Supplementary file 2 [file LSA-2024-02673_TableS2.docx]

**Table S2: Genotyping primers.**

| allele | primer | primer sequence (5′-3′) | Genotype* |
| --- | --- | --- | --- |
| *acr-2(n2420)* | YJ10882 | GGAATATGGGACGTGATTGGTAA | CGCTCTTGTTATGTTCTTGTT |
|  | YJ10883 | ATTATTTCTCTATTGACCGTGGTCC |  |
|  | YJ8874 | GGTATCCTTTCTGGTCGTTTCATC (sequencing primer) |  |
| *eif-3.G(ju807)* | YJ12592 | CCACACATCCAGGAAGGAGCT | TGCCGTCATTACAAAGGAAAC |
|  | YJ12593 | AACGGATTAGTTGCTCGGACGTG |  |
|  | YJ12595 | ATCATTGAGTTCTGCAATTGCACG (sequencing primer) |  |
| *lin-66(ju1661)* | SD20600 | CTTTGGGAACCACGCGGTC | CAAATTTTCAGATGCTCAATG |
|  | SD20601 | GGGACCATAGATTGGTGAGACAATG |  |
|  | SD20602 | GGCTGAACAACAGAGAGCCG (sequencing primer) |  |

***** Red letters indicate nucleotide changes in corresponding alleles.
