# Supplementary material for: C. elegans LIN-66 mediates EIF-3/eIF3-dependent protein translation via a cold-shock domain
Source: Life Sci Alliance. 2024 Jun 17;7(9):e202402673. doi: 10.26508/lsa.202402673 (PMC11184513; doi:10.26508/lsa.202402673)
Supplement: Supplementary file 3 [file LSA-2024-02673_TableS3.docx]

**Table S3: DNA constructs and related primers**

| **construct #** | **transgene(s)** | **primer** | **sequence (5' to 3')** |
| --- | --- | --- | --- |
| pCZGY3547 | *juEx8032/ juEx8033* | YJ12405 | aatttaacaagtcatagctgtttcctggc |
|  |  | YJ12406 | gctgagaattactggccgtcgttttacaac |
|  |  | YJ12407 | gacggccagtaattctcagctacagtacc |
|  |  | YJ12408 | gagactattcatttcgtaagacatcccg |
| pCZGY3556 and pCZGY3557 | *juEx8184/8185[Punc-17B-lin-66C-GFP] and juEx8186/8187*[*Punc-17B-lin-66A-GFP*] | YJ12669 | atgtcttacgaaatgaatagtctcttctcgtc |
|  |  | YJ12670 | tcagttaccaatacggagtgagttcaaac |
|  |  | YJ12767 | ACTCACTCCGTATTGGTAACGCTAGCATGAGTAAAGGAGAAGAACTTTTCACTGG |
|  |  | YJ12768 | CGGTGGCGGCCGCTCTAGAACTAGTtcaTTTGTATAGTTCATCCATGCCATGTG |
| pCZGY3555 | *juEx8190/8191[Plin-66-lin-66C-GFP*] | YJ12669 | atgtcttacgaaatgaatagtctcttctcgtc |
|  |  | SD20603 | GATATCAAGCTTTTTTTCTACCGGTCCCGTTTCAAGCTGTCTTCAGTC |
|  |  | YJ12767 | ACTCACTCCGTATTGGTAACGCTAGCATGAGTAAAGGAGAAGAACTTTTCACTGG |
|  |  | YJ12768 | CGGTGGCGGCCGCTCTAGAACTAGTtcaTTTGTATAGTTCATCCATGCCATGTG |
| pCZGY3583 and pCZGY3584 | *juEx82408241[Plin-66-lin-66C(∆10-44)-GFP*] and *juEx8242/8243[Plin-66-lin-66C(∆10-44 + ∆421-554)-GFP*] | YJ12751 | ATGGGAGCCCCGGACAAC |
|  |  | YJ12752 | GAAGAGACTATTCATTTCGTAAGACATAAGGGC |
| pCZGY3567 and pCZGY3584 | *juEx8194/8195[Plin-66-lin-66C(∆421-554)-GFP*] and *juEx8242/8243[Plin-66-lin-66C(∆10-44 + ∆421-554)-GFP*] | YJ12753 | CAGATTGATCGTTTCTCGCCATCC |
|  |  | YJ12754 | GCGGGTCGCTGCTGGAGTC |
| pCZGY3568 | *juEx8197/8198[Plin-66-lin-66C((∆116-367)-GFP*] | YJ12769 | GAAATGGATATTACTCGTTCCCAGCAG |
|  |  | YJ12770 | AGCCTTGGCAGACAACCAAG |
| pCZGY3578 | *juEx8206/8207[Plin-66-lin-66C(∆298-367)-GFP*] | YJ12769 | GAAATGGATATTACTCGTTCCCAGCAG |
|  |  | YJ12809 | GGCTCCACCACGACGG |
| pCZGY3580 | *juEx8220/8221[Plin-66-lin-66C(∆10-172)-GFP*] | YJ12752 | GAAGAGACTATTCATTTCGTAAGACATAAGGGC |
|  |  | YJ12811 | GGTCCCGATGCTGATGTTCTC |
| pCZGY3582 | *juEx8238/8239[Plin-66-lin-66C(∆2-90-∆387-624)-GFP*] | SD20046 | GTACAAAAAAGCAGGCTCCGCCTAGGATGCCGTCTATGGCTGAACAACAGAGAG |
|  |  | SD20047 | GTTCTTCTCCTTTACTCATGCATGCAATTCCACGTGGTGGCTGTC |
| pCZGY3576 | *juSi395[Peif-3.G::mKate2::eif-3.G]* | YJ12800 | aagtataggaacttcgcatgagggattacaatcaacgaaaggaatattgc |
|  |  | YJ12801 | tccttgatgagctcggacatagctgtaactttactacctgaaaaactattgattatg |
|  |  | YJ12802 | caggtagtaaagttacagctatgtccgagctcatcaaggagaac |
|  |  | YJ12803 | gttgctccacctccacctccacggtgtccgagcttgg |
|  |  | YJ12804 | catccaagctcggacaccgtggaggtggaggtggagcaac |
|  |  | YJ12805 | ttcaaagaaatcgccgacttAaactatgatattttacattggacagaaacccaact |
| pCZGY3602 | *juEx8267/8268[Plin-66-lin-66C(CSD*)-GFP*] | SD20194 | ATCATCGCGGCCTCACTCAAGGACTTTTGTGATCAGATGCTC |
|  |  | SD20195 | CATCTTGTCCTTGCAAGTTGACGCAGCAGCCTTGGCAGACAACCAAG |
|  |  |  |  |
